# Lumazine Peptides from the Marine-Derived Fungus *Aspergillus terreus*

**DOI:** 10.3390/md13031290

**Published:** 2015-03-12

**Authors:** Minjung You, Lijuan Liao, Soo Hyun Hong, Wanki Park, Dah In Kwon, Jeeyeon Lee, Minsoo Noh, Dong-Chan Oh, Ki-Bong Oh, Jongheon Shin

**Affiliations:** 1 Natural Products Research Institute, College of Pharmacy, Seoul National University, San 56-1, Sillim, Gwanak, Seoul 151-742, Korea; E-Mails: lovemin90@snu.ac.kr (M.Y.); lxycxf@snu.ac.kr (L.L.); hong-soo-hyun@hanmail.net (S.H.H.); minsoonoh@snu.ac.kr (M.N.); dongchanoh@snu.ac.kr (D.-C.O.); 2 Department of Agricultural Biotechnology, College of Agriculture and Life Science, Seoul National University, San 56-1, Sillim, Gwanak, Seoul 151-921, Korea; E-Mail: adamas2001@hanmail.net; 3 College of Pharmacy, Seoul National University, Seoul 151-742, Korea; E-Mails: dahin@snu.ac.kr (D.I.K.); jyleeut@snu.ac.kr (J.L.)

**Keywords:** lumazine, peptide, *Aspergillus terreus*, insulin sensitivity

## Abstract

Terrelumamides A (**1**) and B (**2**), two new lumazine-containing peptides, were isolated from the culture broth of the marine-derived fungus *Aspergillus terreus*. From the results of combined spectroscopic and chemical analyses, the structures of these compounds were determined to be linear assemblies of 1-methyllumazine-6-carboxylic acid, an amino acid residue and anthranilic acid methyl ester connected by peptide bonds. These new compounds exhibited pharmacological activity by improving insulin sensitivity, which was evaluated in an adipogenesis model using human bone marrow mesenchymal stem cells. In addition, the compounds exhibited fluorescence changes upon binding to DNA, demonstrating their potential applications to DNA sequence recognition.

## 1. Introduction

Fungi from marine environments are widely recognized as emerging sources of biologically active and structurally unique secondary metabolites [1,2,3,4,5]. Although studies on these organisms began considerably later than those on their counterparts from terrestrial environments, significant numbers of metabolites have been found annually since the late 1990s [6]. This trend has accelerated in recent years due to both the demand for the production of mass bioactive compounds and the technical progress in related fields, such as microbial genetics and bioinformatics [7]. Therefore, along with bacteria originating from the same environments, marine-derived fungi are considered to be a new frontier for natural products research.

**Figure 1:**
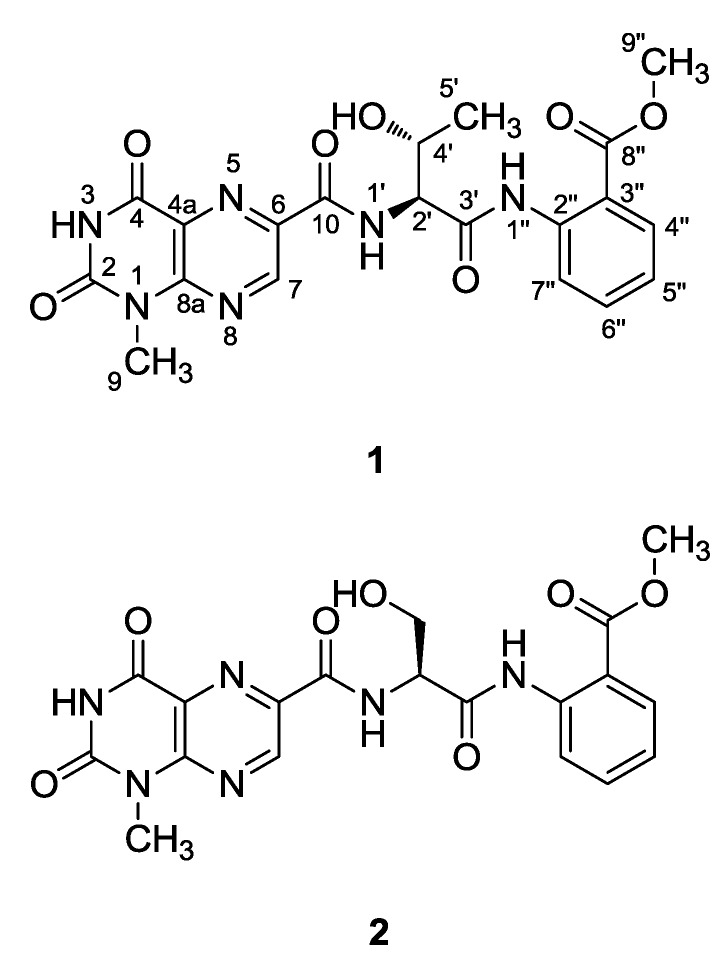
Structures of compounds **1**–**2**.

In our search for novel bioactive compounds from marine fungi, a strain of *Aspergillus terreus* was collected from marine sediment from Jeju Island, Korea, and the organic extract of this fungi exhibited mild cytotoxicity (IC_50_ 370 μg/mL) against the human leukemia K562 cell line. More importantly, the ESI-LC/MS (liquid chromatography coupled with electrospray ionization mass spectrometry) iniprofile of the extract revealed the presence of novel constituents which motivated us to investigate its metabolites in detail. A large-scale solid-substrate culture of the strain, followed by extraction and chromatographic separation, led to the isolation of two new metabolites, in addition to several known metabolites, such as the alkaloids acetylaszonalenin and asterrelenin [8,9], the meroterpenoids territrems A and B [10], and the pyridine-containing dihydroisoflavipucine [11]. Here, we report the structural determinations of terrelumamides A (**1**) and B (**2**) (Figure 1), which are new linear lumazine peptides. These compounds are structurally related to penilumamides A–D, which were recently isolated from marine-derived *Penicillium* sp. and *Aspergillus* sp. Fungi [12,13]. However, to the best of our knowledge, the 1-methyllumazine-6-carboxylic acid moiety of these terrelumamide compounds is the first such example among natural products. These compounds exhibited the pharmacological activity to improve the insulin sensitivity, which was evaluated in a cell-based model for adipogenesis using human bone marrow mesenchymal stem cells (hBM-MSCs).

## 2. Results and Discussion

**Figure 2:**
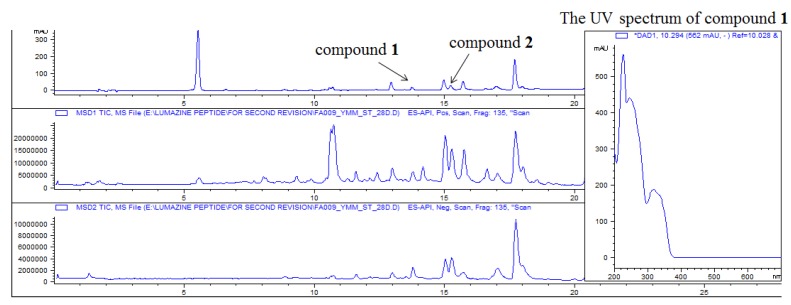
The ESI-LC/MS profile of crude extract.

**Figure 3:**
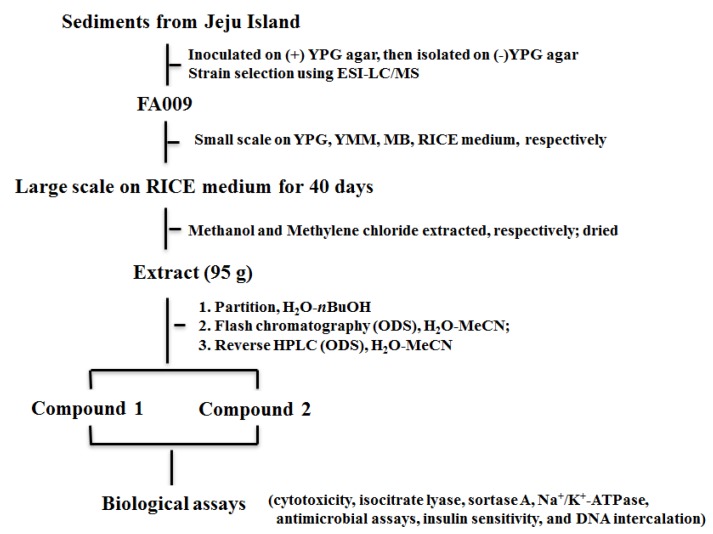
The work flow sheet of FA009.

The crude extract from this fungal strain was analyzed using ESI-LC/MS which was shown in Figure 2. And the procedure from marine sediments collection to bioactive compounds was also shown in Figure 3. The molecular formula of terrelumamide A (**1**) was determined to be C_20_H_20_O_7_N_6_ based on HRFABMS analysis. The highly unsaturated nature of this compound was indicated by the fourteen degree of unsaturation present in the mass data, and by the presence of signals from fifteen carbons in the aromatic and carbonyl regions in the ^13^C NMR data. This interpretation was consistent with the strong absorption bands at 1708 and 1690 cm^−1^ in the IR spectrum. The presence of several absorption maxima at 220–340 nm (E band) in the UV spectrum also supported the presence of an aromatic moiety. The remaining carbons in the ^13^C NMR data were two methines and three methyl carbons in the upfield region (Table 1).

**Table 1:** NMR Data of Compounds **1** and **2** in DMSO-*d_6_*.

1				2		
Position	δ_C_, Type	δ_H_, Mult (*J* in Hz)	HMBC ^a^	δ_C_, Type	δ_H_, Mult (*J* in Hz)	HMBC
2	150.0, C			150.0, C		
3-NH		12.18, s	4, 4a		12.15, br s	
4	159.3, C			159.3, C		
4a	127.2, C			127.1, C		
6	138.2, C			138.5, C		
7	146.2, CH	9.30, s	4a, 6, 8a	146.3, CH	9.28, s	6, 8a
8a	151.1, C			151.0, C		
9	28.6, CH_3_	3.52, s	2, 8a	28.5, CH_3_	3.52, s	2, 8a
10	162.7, C			162.6, C		
1′-NH		8.51, d (8.0)	10, 2′, 4′		8.75, d (7.5)	10, 2′, 4′
2′	59.7, CH	4.56, dd (8.0, 2.8)	3′, 4′	56.6, CH	4.66, ddd (7.5, 5.0, 5.0)	3′, 4′
3′	168.8, C			168.7, C		
4′	65.9, CH	4.42, m		61.0, CH_2_	4.01, ddd (10.6, 5.0, 5.0)	2′, 3′
					3.89, ddd (10.6, 5.0, 5.0)	2′, 3′
4′-OH		5.58, d (4.8)	2′, 4′, 5′		5.44, dd (5.0, 5.0)	2′, 4′
5′	20.5, CH_3_	1.19, d (6.3)	2′, 4′			
1″-NH		11.11, s	3′, 3″, 7″		11.11, s	3′, 3″, 7″
2″	139.2, C			139.3, C		
3″	117.1, C			117.0, C		
4″	130.6, CH	7.92, d (8.0)	2″, 6″, 8″	130.6, CH	7.92, d (8.0)	2″, 6″, 8″
5″	123.4, CH	7.21, dd (8.0, 8.0)	3″, 7″	123.3, CH	7.21, dd (8.0, 8.0)	3″, 7″
6″	134.1, CH	7.64, dd (8.0, 8.0)	2″, 4″	134.1, CH	7.63, dd (8.0, 8.0)	2″, 4″
7″	120.7, CH	8.44, d (8.0)	3″, 5″	120.7, CH	8.44, d (8.0)	3″, 5″
8″	167.3, C			167.3, C		
9″	52.4, CH_3_	3.70, s	8″	52.3, CH_3_	3.70, s	8″

^a^ HMBC correlations are started from the proton (s) to the indicated carbon.

**Figure 4:**
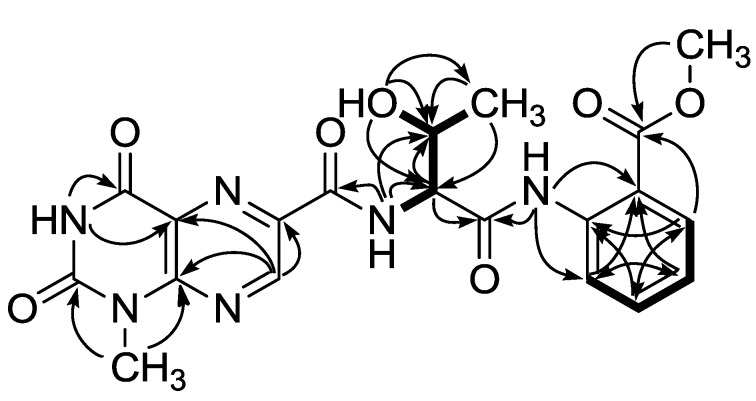
The COSY (bold line) and selected HMBC (arrows) correlations of compound **1**.

Given this information, the structure of compound **1** was determined through combined 2-D NMR experiments. First, the HSQC data revealed the direct attachment of an isolated methine proton at δ_H_ 9.30 to the carbon at δ_C_ 146.2. The long-range carbon-proton correlations of this proton with the quaternary carbons at δ_C_ 151.1, 138.2 and 127.2 in the HMBC data placed these carbons at the neighboring positions. Among these carbons, the one at δ_C_ 127.2 exhibited an additional correlation with an exchangeable proton at δ_H_ 12.18, which also correlated with a quaternary carbon at δ_C_ 159.2. The chemical shifts of a methyl carbon at δ_C_ 28.6 and its protons at δ_H_ 3.52 revealed the presence of an *N*-methyl group of a heteroaromatic moiety. The neighboring positions of this methyl group were occupied by the quaternary carbons at δ_C_ 151.1 and 150.0 deduced from the HMBC data. Taken together, both the chemical shifts of the protons and carbons and the HMBC correlations among them revealed the presence of a 1-methyllumazine moiety, which was confirmed by comparing of the spectroscopic data with those of lumazine analogs in the literature (Figure 4) [14,15]. Based on the combined HMBC data, the *N*-methyl group was placed at the N-1 position.

The ^1^H-^1^H COSY data revealed a spin system that consisted of the protons at δ_H_ 8.51 (1 H, d, *J* = 8.0 Hz, exchangeable), 5.58 (1 H, d, *J* = 4.8 Hz, exchangeable), 4.56 (1 H, dd, *J* = 8.0, 2.8 Hz), 4.42 (1 H, m), and 1.19 (3 H, d, *J* = 6.3 Hz). Aided by the HSQC and HMBC analyses, these protons and their attached carbons were readily assigned to a threonine (Thr) amino acid residue. The carbonyl carbons of this residue and neighboring unit were found at δ_C_ 168.8 and 162.7 based on their HMBC correlations with the NH and α-carbonyl protons at δ_H_ 5.58 and 4.56, respectively (Figure 4). However, the exact assignments of these carbonyl carbons were not accomplished at this stage due to the lack of additional HMBC correlations with the Thr protons.

The ^13^C NMR data of **1** indicated that six carbons were present in the δ_C_ 139.2–117.1 region, which suggested the presence of a benzene moiety (Table 1). The chemical shifts of the attached protons at δ_H_ 8.44–7.21 and the mutual spin couplings among these protons, as well as the large (*J* = 8.0 Hz) vicinal coupling constants, were indicative of a 1,2-disubstituted benzene, which was confirmed by the HMBC data (Figure 4). Additional correlations of the aromatic proton at δ_H_ 7.92 (H-4″) and a methyl proton at δ_H_ 3.70 (H-9″) with a carbonyl carbon at δ_C_ 167.3 (C-8″) confirmed the attachment of a methyl carboxylate group as a substituent of the benzene. Similarly the correlations of a NH proton at δ_H_ 11.11 (1″-NH) with the aromatic carbons at δ_C_ 120.7 (C-7″) and 117.1 (C-3″) placed the amine group as the adjacent substituent, thus constructing an anthranilic acid methyl ester moiety.

**Figure 5:**
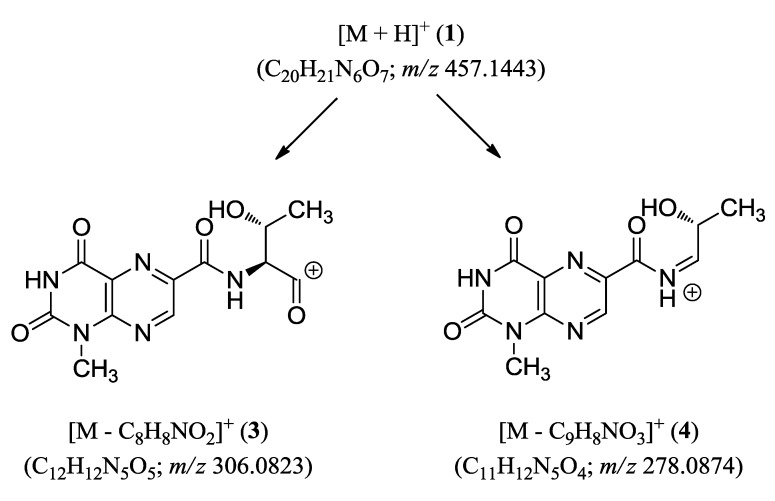
ESI-Q-TOF-MS/MS fragmentations of compound **1**.

The connectivity among the partial structures was also determined through an HMBC analysis. The two carbonyl carbons at δ_C_ 168.8 and 162.7 correlated to the Thr protons, and the additional correlation between the former carbon and the 1″-NH of anthranilic ester not only linked these moieties but also assigned this carbon as the carbonyl (C-3′) of the Thr residue. Accordingly, the remaining carbonyl carbon at δ_C_ 162.7 (C-10) must belong to the neighboring unit, 1-methyllumazine carboxylic acid, despite its lack of long-range correlations with the H-7 or other protons of the lumazine moiety. This interpretation was supported by the ESI-Q-TOF-MS/MS (electrospray ionization quadrupole time-of-flight mass spectrometry) analysis in which fragments consisting of the lumazine carboxylic acid-Thr were analyzed with high-resolution (Figure 5).

Terrelumamide A (**1**) possesses two stereogenic centers at its Thr residue. The l-configuration at the α-stereogenic center was assigned based on an advanced Marfey’s analysis, in which the l-FDAA adduct of the hydrolysate presented a shorter HPLC retention time than the d-FDAA adduct (Experimental Section) [16,17]. Similarly, the *R*-configuration was also assigned for the β-stereogenic center on the basis of Marfey’s analysis, in which the l-FDAA adduct of the hydrolysate presented a retention time that were identical to those of the adduct derived from the authentic l-Thr, whereas a significant difference was found with respect to the *allo*-l-Thr adduct. Thus, the structure of terrelumamide A (**1**) was determined to be a new lumazine peptide.

A review of the literature revealed that alkylated lumazine derivatives have been found from a number of terrestrial and marine animals. Simple lumazine-containing compounds from marine animals include those from the sponges *Leucetta microraphis* [18], *Corallistes fulvodesmus* [19], *Corallistes undulates* [14], and *Clathria* sp. [20], the polychaete *Odontosyllis undecimdonta* [21,22,23], and the ascidian *Leptoclinides durust* [24], whereas those containing the lumazine-6-carboxylic acid moiety were also found in the freshwater leech *Limnatis nilotica* [15]. Unlike the alkylated lumazines of animal origin, the recently reported penilumamides A–D from marine-derived *Penicillium* sp. and *Aspergillus* sp. fungi are structurally distinct, which is primarily due to their peptide nature and anthranilic methyl ester [12,13]. Because terrelumamide A (**1**) contains a lumazine moiety, an amino acid residue and a methyl anthranilic ester, it is structurally related to these fungi-derived lumazine peptides. However, the 1-methyllumazine-6-carboxylic acid and the Thr unit both provide structural novelty to **1**. Furthermore, the 1-methyllumazine carboxylic acid either as a monomer or as a partial structural moiety of a compound has not been found in natural products. Our discovery of an additional lumazine peptide may provide further evidence for the biogenetic distinction between marine-derived fungi and animals.

The molecular formula of terrelumamide B (**2**) was established to be C_19_H_18_O_7_N_6_ based on an HRFABMS analysis. The ^13^C and ^1^H NMR data of this compound were very similar to those of **1**. A detailed examination of the ^13^C NMR data revealed that the 1-methyllumazine-6-carboxylic acid and anthranilic acid methyl ester moieties of **1** were also present in **2**. The most noticeable difference occurred at the amino acid unit, in which the C-4′ and C-5′ of Thr were replaced with a methylene carbon at δ_C_ 61.0. Corresponding differences were also observed in the ^1^H NMR data; the signals of the H-4′ methine and H-5′ of **1** were replaced with those of methylene protons at δ_H_ 4.01 and 3.89 (Table 1). These spectroscopic changes were readily explained by the replacement of Thr of **1** with a serine (Ser) residue in **2**, which was confirmed by combined 2-D NMR analyses. For **2**, the absolute configuration of the α-stereogenic center of Ser was assigned to be L through an advanced Marfey’s analysis, in which the l-FDAA adduct of the hydrolysate presented a clearly shorter HPLC retention time than the d-FDAA adduct. Thus, the structure of terrelumamide B (**2**) was determined to be a lumazine peptide containing an l-Ser residue.

The lumazine monomer and its synthetic *N*-alkylated derivatives have been reported to inhibit the growth of methanogens and formantion of methane [25], and the production of tumor necrosis factor-α [26], respectively. Despite remarkable structural variations, however, more complex compounds that contain lumazine moieties do not possess significant bioactivity, and the only reported bioactivity was a weak inhibitory activity against *E. coli* at 50 μg/disk in a disk diffusion assay [20]. The recently reported penilumamides A–D obtained from marine-derived fungi were also inactive in various bioactivity assays such as cytotoxicity, antimicrobial and antiviral assays, and cellular Ca^++^ signaling activity tests [12,13]. These findings are consistent with our measurements, in which the terrelumamides were inactive against the K652 and A549 cell lines (IC_50_ > 100 μM) and selected strains of Gram-positive and Gram-negative bacteria and pathogenic fungi (MIC > 100 μg/mL). These compounds were also inactive (IC_50_ > 100 μM) against various enzymes, such as sortase A, isocitrate lyase and Na^+^/K^+^-ATPase.

**Figure 6:**
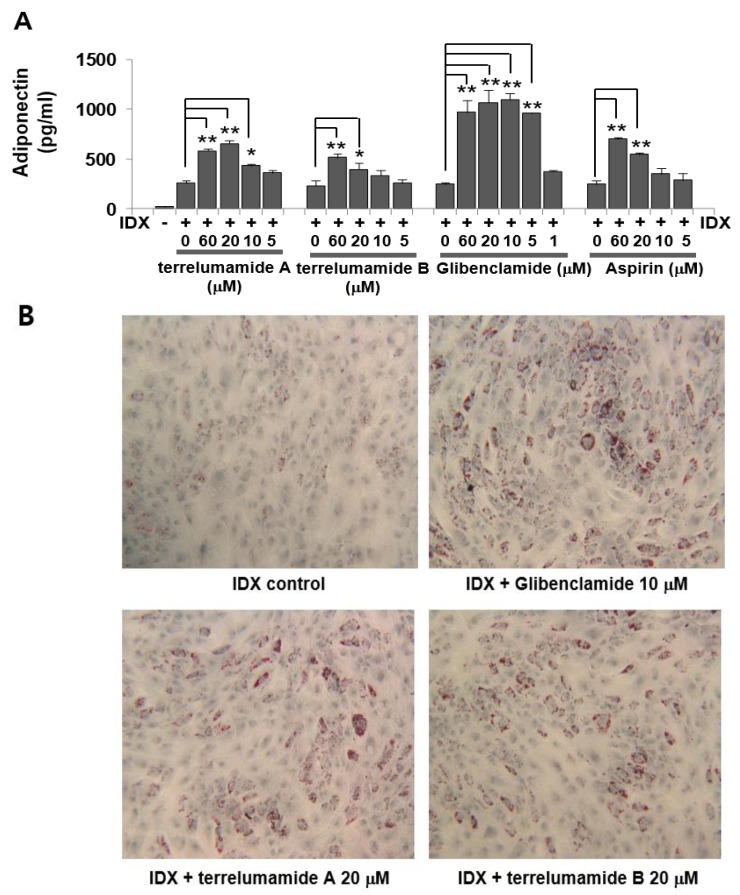
(**A**) Effects of terrelumamides A and B on the production of adiponectin during adipogenesis in human bone marrow mesenchymal stem cells (hBM-MSCs). After the induction of adipogenesis, the culture medium was changed every two days. On the 8th day of culture, the cell culture supernatants were harvested. ELISAs were performed to measure the levels of adiponectin that accumulated in the cell culture supernatants over the 48 h after the last medium exchange. Glibenclamide was used as the positive control. The values represent the mean ± SD (*n* = 3). * *P* ≤ 0.05 and ** *P* ≤ 0.01; (**B**) Phenotypic changes in the hBT-MSCs. Eight days after adipogenic stimulation with IDX, the lipid droplets in the adipocytes were stained with Oil Red O (ORO).

In contrast, using a cell-based assay to evaluate anti-diabetic compounds, we observed that the terrelumamides increased the production of adiponectin during adipogenesis in hBM-MSCs (Figure 6). The level of adiponectin production in the adipogenesis model of hBM-MSCs has been used as a measure of insulin sensitivity [27]. In this study, glibenclamide and aspirin were used as dual positive controls because their pharmacological mechanisms for improving insulin sensitivity are different [28]. Glibenclamide achieves antidiabetic activity by binding with sulfonylurea receptor 1 (SUR1), which inhibits the conductance of the adenosine triphosphate (ATP)-dependent potassium (K_ATP_) channel whereas aspirin inhibits the serine kinase IKKβ [28]. The pharmacological activity of glibenclamide reached a plateau at concentrations greater than 5 μM (Figure 6). Although aspirin significantly increased the production of adiponectin, the maximum effect achieved by aspirin in the hBM-MSCs was less than that of glibenclamide (Figure 6). Based on the maximum pharmacological activity of glibenclamide, the median effective concentration (EC_50_) values of glibenclamide and aspirin were 3.47 and 145.6 μM, respectively. The EC_50_ values of terrelumamides A (**1**) and B (**2**) were 37.1 and 91.9 μM, respectively. In addition, the maximum increase in adiponectin levels induced by terrelumamide A was 56.9% relative to that generated by glibenclamide. Therefore, the terrelumamides in this study were more potent than aspirin but less potent than glibenclamide. Given the possibility of producing diverse structural variations through chemical synthesis, our findings may contribute to the development of a novel platform of insulin-related therapeutic agents.

**Figure 7:**
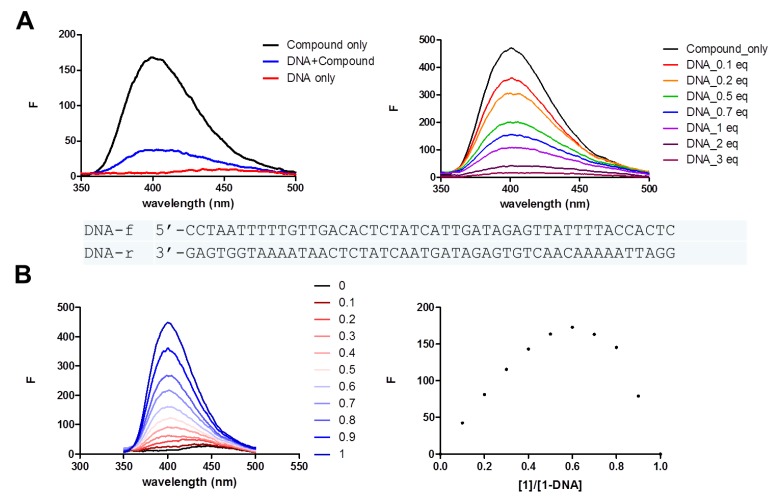
(**A**) Fluorescence spectra of compound (**1**) (2 μM) upon addition of DNA duplex; (**B**) The Job plot obtained from the fluorescence response upon DNA binding.

In order to see the compound’s potentials as anticancer agents or gene expression modulators, we also explored the DNA-binding properties of lumazine peptides using fluorescence spectroscopy. Figure 7 demonstrates the fluorescence decrease upon adding double-stranded DNA oligomers containing sequences from the *tetO* promoter region. The terrelumamide A (**1**) exhibited strong emission peak at around 400 nm by exciting at 260 nm, which gradually decreased upon addition of DNA duplex. The Job plot analyzed from the fluorescence spectra of the titration suggests that the stoichiometry of the compound-DNA complex is 2:1, indicating two binding sites within the 48 bp DNA sequence*.* Based on the stoichiometry we observed, it appears to have a combined binding mode; intercalation by terrelumamide A along with groove binding by the peptide backbone. The results may suggest a new direction to explore biological activities of lumazine peptides.

## 3. Experimental Section

### 3.1. General Experimental Procedures

Optical rotations were measured on a JASCO P-1020 polarimeter (Jasco, Tokyo, Japan) using a 1 cm cell. UV spectra were acquired with a Hitachi U-3010 spectrophotometer (Hitachi High-Technologies, Tokyo, Japan). IR spectra were recorded on a JASCO 4200 FT-IR spectrometer (Jasco, Tokyo, Japan) using a ZnSe cell. NMR spectra were recorded in DMSO-*d*_6_ solutions on Bruker Avance 600 and 500 spectrometers (Bruker, Billerica, MA, USA). Proton and carbon NMR spectra were measured at 600 and 150 MHz (**1**) or 500 and 125 MHz (**2**), respectively (See Supplementary Information, Figures S1–S5 and S7–S11). High-resolution FAB mass spectrometric data were obtained at the Korea Basic Science Institute (Daegu, Korea) and were acquired using a JEOL JMS 700 mass spectrometer (Jeol, Tokyo, Japan) with *meta*-nitrobenzyl alcohol (NBA) as a matrix for the FABMS. Low-resolution ESIMS data were recorded on an Agilent Technologies 6130 quadrupole mass spectrometer (Santa Clara, CA, USA) coupled to an Agilent Technologies 1200 series HPLC (Santa Clara, CA, USA). The ESI-Q-TOF MS/MS measurements were performed on an Agilent Technologies 6530 Accurate-Mass Q-TOF LC/MS spectrometer (Santa Clara, CA, USA) with an Agilent Technologies 1260 series HPLC (Santa Clara, CA, USA) (See Supplementary Information, Figures S6). Semi-preparative HPLC was performed on a Spectrasystem p2000 (Thermo, Waltham, MA, USA) equipped with a refractive-index detector (Spectrasystem RI-150) and an YMC ODS-A column (10 × 250 mm). All solvents used were spectroscopic grade or were distilled prior to use.

### 3.2. Isolation and Identification of the Fungal Strain

The fungal strain *Aspergillus* *terreus* (strain number FA009) was isolated from marine sediments collected offshore of Jeju Island, Korea, in October, 2012. FA009 was identified using standard molecular biological protocols by DNA amplification and sequencing of the ITS region. Genomic DNA extraction was performed using Intron’s i-genomic BYF DNA Extraction Mini Kit according to the manufacturer’s protocol. The nucleotide sequence of FA009 was deposited in the GenBank database under accession number KF146985. The 18S rDNA sequence of this strain exhibited 100% identity with that of *Aspergillus terreus* KAML04 (GenBank accession number KF146985).

### 3.3. Fermentation and Isolation

The fungal strain was cultured on solid YPG media (5 g of yeast extract, 5 g of peptone, 10 g of glucose, 16 g of agar, and 24.8 g of Instant Ocean in 1 L of distilled water) for 7 days. An agar plug (1 cm × 1 cm) was inoculated in a 250 mL flask that contained 100 mL of YPG media for 7 days. Then, 10 mL of each culture was transferred to a 2.8 L Fernbach flask that contained rice media (200 g of rice, 0.5 g of yeast extract, 0.5 g of peptone, and 12.4 g of Instant Ocean in 500 mL of distilled water). In total, 600 g of rice media was prepared and cultivated for 40 days at 28 °C, with stirring once a week. The entire culture was macerated and repeatedly extracted with MeOH (2 L × 2) and CH_2_Cl_2_ (2 L × 2), and then the solvent was evaporated *in vacuo* to yield 95 g of extract. The extracts were partitioned between H_2_O (68 g) and *n*-BuOH (27 g), and the latter fraction was separated by C_18_ reversed-phase vacuum flash chromatography using a sequential mixture of H_2_O and MeCN as the eluents (five fractions in a H_2_O–MeCN, gradient from 90:10 to 50:50), methanol, acetone, and finally EtOAc. Based on the ^1^H NMR analysis results, the fractions eluted with H_2_O–MeCN (60:40) (920 mg) were separated by semi-preparative reversed-phase HPLC (H_2_O–MeCN, 70:30, 2.0 mL/min) to afford 18 peaks. Peaks 16 and 12 were further purified by reversed-phase HPLC (H_2_O–MeOH, 53:47, 2.0 mL/min), affording compounds **1** (14.5 mg) and **2** (1.9 mg), respectively.

Terrelumamide A (**1**): White amorphous solid; (α)25D +94.5 (*c* 0.25, MeOH); UV (MeOH) λ_max_ (log ε) 222 (4.43), 248 (4.28), 274 (4.11), 316 (3.87), 336 (3.82) nm; IR (ZnSe) ν_max_ 3726, 3600, 1708, 1690, 1516 cm^−1^; ^1^H and ^13^C NMR data, see Table 1; HRFABMS, *m/z* 457.1475 (M + H)^+^ (calcd for C_20_H_21_O_7_N_6_, 457.1472).

Terrelumamide B (**2**): White amorphous solid, (α)*25D* +103.7 (c 0.25, MeOH); UV (MeOH) λ_max_ (log ε) 222 (4.43), 248 (4.24), 274 (4.06), 316 (3.85), 336 (3.79) nm; IR (ZnSe) ν_max_ 3728, 3341, 2963, 1708, 1690, 1523 cm^−1^; ^1^H and ^13^C NMR data, see Table 1; HRFABMS, *m/z* 443.1313 (M + H)^+^ (calcd for C_19_H_19_O_7_N_6_, 443.1315).

### 3.4. Determination of the Amino Acid Absolute Configurations [16,17]

Compound **1** (0.5 mg) was dissolved in 0.5 mL of 6 N HCl and hydrolyzed at 110 °C for 2 h. The solvent and traces of HCl were removed by the addition of 0.5 mL of distilled water and evaporation *in vacuo*. To the divided hydrolysate (0.25 mg each), 100 μL of 1 N NaHCO_3_ and 50 μL of 1% l- and d-FDAA (1-fluoro-2,4-dinitrophenyl-5-l(or d)-alanine amide) in acetone were added. The solution was heated at 80 °C for 3 min. After the reaction, 50 μL of 2 N HCl was added to the mixture to neutralize the mixture, followed by the addition of 300 μL of a 50% aqueous MeCN solution. The FDAA derivatives of threonine were analyzed by ESI-LC/MS (H_2_O–MeCN, from 90:10 to 30:70 containing 0.1% formic acid over 40 min using a C_18_ reversed-phase column 100 × 4.6 mm, 0.7 mL/min). The retention times of the l- and d-FDAA-derivatized hydrolysates were 14.9 and 17.3 min, respectively, indicating the l-configuration of the α-stereogenic center of the Thr residue of **1**.

To assign a configuration to the additional β-stereogenic center of Thr, the authentic samples of l-Thr and *allo*-l-Thr were derivatized with l-FDAA and their retention times were compared to those of l-FDAA-derivatized hydrolysates of **1**. The retention times of each FDAA-derivative of l-Thr, *allo*-l-Thr, and the hydrolysates were 15.1, 19.0, and 15.2 min, respectively, as determined by ESI-LC/MS analysis (H_2_O–MeOH, 60:40 containing 0.1% formic acid, over 35 min using a C_18_ reversed-phase column 250 × 4.6 mm, 1.0 mL/min). Thus, the absolute configuration of threonine was determined to be l-Thr.

The Ser residue of **2** was also analyzed using an advanced Marfey’s method with the same protocol as that used for **1**. The HPLC retention times of the l- and d-FDAA-derivatized hydrolysates were 14.0 and 14.7 min, respectively, indicating an l-Ser configuration for **2**.

The results were enclosed in Supplementary Information (Figures S2–S15).

### 3.5. Biological Assays

The cytotoxicity assays were performed in accordance with protocols reported in the literature [29,30]. Isocitrate lyase, sortase A, Na^+^/K^+^-ATPase, and antimicrobial assays were performed according to previously reproted methods [31,32,33,34]. The insulin sensitivity test based on an adipogenesis model using hBM-MSCs was performed as previously reported [27].

### 3.6. Fluorescence Measurements

48 bp DNA oligomers were purchased from Cosmogenetech (Seoul, Korea). Equimolar amounts of single-stranded DNA samples were annealed to form the duplex by mixing both oligomers in water at 95 °C for 5 min followed by cooling down to room temperature. Fluorescence emission changes upon DNA binding were measured using a JASCO FP-6500 spectrofluorometer (Jasco, Tokyo, Japan). All measurements were performed in 20 mM Tris buffer (pH 7.5) containing 100 mM NaCl.

## 4. Conclusions

Two new lumazine peptides, terrelumamides A (**1**) and B (**2**), were isolated from the marine-derived fungus *Aspergillus terreus*. These compounds possessed structural novelty at their 1-methyllumazine-6-carboxylic acid units, which is unprecedented as a component of natural products. The l-Thr (**1**) and l-Ser (**2**) units further distinguished these compounds from penilumamides from other marine-derived fungi. Importantly, this study is the first discovery of the enhancement effect of lumazine-containing natural products on insulin sensitivity. Fluorescence spectroscopy also demonstrates that the terrelumamide A binds to DNA duplex, suggesting new applications of lumazine peptides in DNA sequence recognition.

## Supplementary Files

Supplementary File 1
