# Supplementary material for: Lumazine Peptides from the Marine-Derived Fungus Aspergillus terreus
Source: Mar Drugs. 2015 Mar 12;13(3):1290–303. doi: 10.3390/md13031290 (PMC4377984; doi:10.3390/md13031290)
Supplement: Supplementary File 1 [file marinedrugs-13-01290-s001.pdf]

Supplementary Information

Table of Content

**Fungal Strain Isolation**	
**Biological Assays**	
The ^{1}H NMR (300 MHz, DMSO-d_6) spectrum of terrelumamide A (**1**)	Figure S1
The ^{13}C NMR (75 MHz, DMSO-d_6) spectrum of terrelumamide A (**1**)	Figure S2
The HSQC (500 MHz, DMSO-d_6) spectrum of terrelumamide A (**1**)	Figure S3
The COSY (500 MHz, DMSO-d_6) spectrum of terrelumamide A (**1**)	Figure S4
The HMBC (500 MHz, DMSO-d_6) spectrum of terrelumamide A (**1**)	Figure S5
The ESI-Q-TOF-MS/MS spectrum of terrelumamide A (**1**)	Figure S6
The ^{1}H NMR (500 MHz, DMSO-d_6) spectrum of terrelumamide B (**2**)	Figure S7
The ^{13}C NMR (125 MHz, DMSO-d_6) spectrum of terrelumamide B (**2**)	Figure S8
The HSQC (600 MHz, DMSO-d_6) spectrum of terrelumamide B (**2**)	Figure S9
The COSY (600 MHz, DMSO-d_6) spectrum of terrelumamide B (**2**)	Figure S10
The HMBC (600 MHz, DMSO-d_6) spectrum of terrelumamide B (**2**)	Figure S11
The results of advanced Marfey's method of terrelumamide A (**1**)	Figure S12
The results of advanced Marfey's method of terrelumamide B (**2**)	Figure S13
The workflow sheet of extraction procedure	Figure S14
The LC chart of isolation	Figure S15

S1. Fungal Strain Isolation

First, we dried the sediment overnight without sterilization. Then spread and inoculated the sediment on the prepared agar petri dishes with anti-bacteria YPG medium (150 mg of penicillin G sodium salt, 150 mg of streptomycin sulfate salt, 4 g of yeast extract, 4 g of peptone, 10 g of glucose, 16 g of agar, and 24.8 g of sea salt in 1 L of distilled water). Growth on petri dishes were performed on 28 ℃. After inoculation, plates were regularly examined in order to verify the growth of filamentous fungi. Pure fungal strains were obtained by successive purification steps. And the pure strains were inoculated on YPG medium (4 g of yeast extract, 4 g of peptone, 10 g of glucose, 16 g of agar, and 24.8 g of sea salt in 1 L of distilled water).

S2. Biological Assays

S2.1. Cytotoxicities and Anti-Microbial Assays

Cytotoxicity assays. The cytotoxicities were performed toward the leukemia cell line K562 and lung cancer cell line A549. The cells were cultured in DMEM supplemented with penicillin G (100 U/mL), streptomycin (100 μg/mL), L-glutamin, and 10% fetal calf serum at 37 ℃ in a humidified atmosphere containing 5% CO_2. Compound **1** and **2** were prepared in DMSO. Each stock solution was diluted with DMEM to prepare serial ten-fold dilution from 100 to 1 μg/mL. A549 cells were seeded at the density of 2000 cells per well of 96-well plates in 100 μL. After 12 h incubation, 100 μL of three concentrations of **1** and **2** were added to the A549. K562 cells were seeded at the density of 2000 cells per well of 96-well plates in 100 μL and treated compound **1** and **2** directly. The cells treated only DMSO were

used as control and treated only medium without any drug were used as negative control. After 48 h incubation, 25 μL of MTT solution was added to each well and incubated for 4 h at 37 ℃. Then the suction of the medium was performed using pump and 100 μL solubilizing buffer (10% sodium dodecyl sulfate dissolve in 0.01 N HCl) was added to each well.

Anti-bacterial assays. Three Gram-negative bacteria (*Escherichia coli* ATCC 35270, *Proteus vulgaris* ATCC 3851 and *Salmonella typhimurium* ATCC 14028) and three Gram-positive bacteria (*Bacillus subtilis* ATCC 6633, *Micrococcus luteus* IFO 12708 and *Staphylococcus aureus* ATCC 6538p) were used for antimicrobial activity tests. Bacteria were grown overnight in Luria Bertani (LB) broth at 37 ℃, harvested by centrifugation, and then washed twice with sterile distilled water. Stock solutions of **1** were prepared in DMSO. Each stock solution was diluted with Standard method broth (Difco, Sparks, MD, USA) to prepare serial two-fold dilutions in the range of 100 to 0.8 μg/mL. Ten microliters of the broth containing about 10^5 colony-forming units (cfu)/mL of test bacteria was added to each well of 96-well microtiter plate. Culture plates were incubated for 24 h at 37 ℃.

Anti-fungal assays. *Aspergillus fumigatus* (HIC 6094), *Trichophyto rubrum* (IFO 9185), *T. mentagrophytes* (IFO 40996), and *Candida albicans* (ATCC 10231) were used for antifungal activity tests. *C. albicans* was grown for 48 h at 28 ℃ in YPD broth (1% yeast extract, 2% peptone, and 2% dextrose), harvested by centrifugation, and then washed twice with sterile distilled water. *A. fumigatus*, *T. rubrum*, and *T. mentagrophytes* were plated in potato dextrose agar (PDA) (Difco, Sparks, MD, USA) and incubated at 28 ℃ for 2 weeks. Spores were washed three times with sterile distilled water and resuspended in distilled water to obtain an initial inoculum size of 10^5 spores/mL. Compound **1** was dissolved in DMSO and diluted with potato dextrose broth (Difco, Sparks, MD, USA) to prepare serial two-fold dilutions in the range of 100 to 0.8 μg/mL. Ten microliters of the broth containing about 10^3 (for yeast) and 10^4 (for filamentous fungi) cells/mL of test fungi was added to each well of a 96-well microtiter plate. Culture plates were incubated for 48–72 h at 28 ℃.

S2.2. Insulin Sensitivity Assay with the Adipogenesis Model in hBM-MSCs

hBM-MSCs were purchased from Lonza, Inc. (Walkersville, MD, USA). hBM-MSCs were maintained and differentiated into adipocytes according to the manufacturer's instructions with minor modifications [1]. Briefly, hBM-MSCs were maintained in Dulbecco-modified eagle's medium (DMEM) with low glucose (1 g/L glucose) containing 10% fetal bovine serum (FBS) (Lonza, Walkersville, MD, USA) at 37 ℃ in a humidified cell culture incubator under 5% CO_2. To induce adipogenesis, hBM-MSCs were grown to 100% confluence. After the confluence, the growth medium was exchanged with DMEM with high glucose (4.5 g/L glucose) supplemented with 10% FBS, 10 μg/ml insulin, 1 μM dexamethasone and 0.5 mM 3-isobutyl-1-methylxanthine (IBMX) (the IDX condition). The test compound was added with the IDX condition to examine the activity. After the induction of adipogenesis, media was exchanged with the adipogenesis condition at every 48 h for three times. After the last medium exchange, cell culture supernatants were harvested for enzyme-linked immunosorbent assay (ELISA) to measure adiponectin concentration. Adiponectin ELISA was performed with the Quantikine™ adiponectin immunoassay kit (R&D Systems, Minneapolis, MN, USA). Adipogenesis was also assessed by Oil Red O staining by a method previously described [1]. To visualize the nucleus, hBM-MSCs were counterstained with hematoxylin reagent for 2 min and then washed

twice with H_2O. The differentiated adipocytes were photographed using an Olympus IX71 inverted phase-microscope (Olympus Co., Tokyo, Japan).

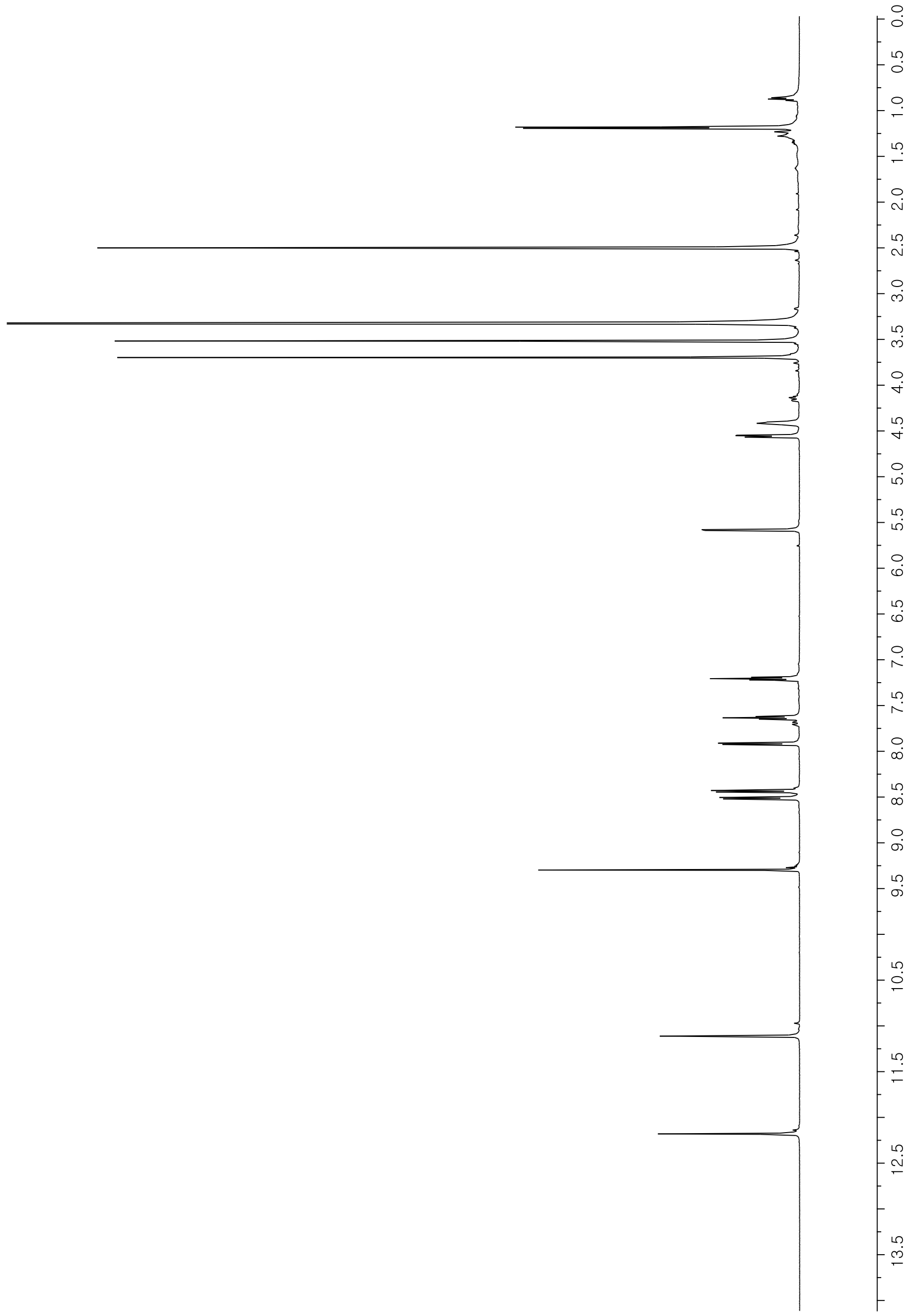

Figure S1. The ^{1}H NMR (600 MHz, DMSO-d_6) spectrum of terrelumamide A (**1**).

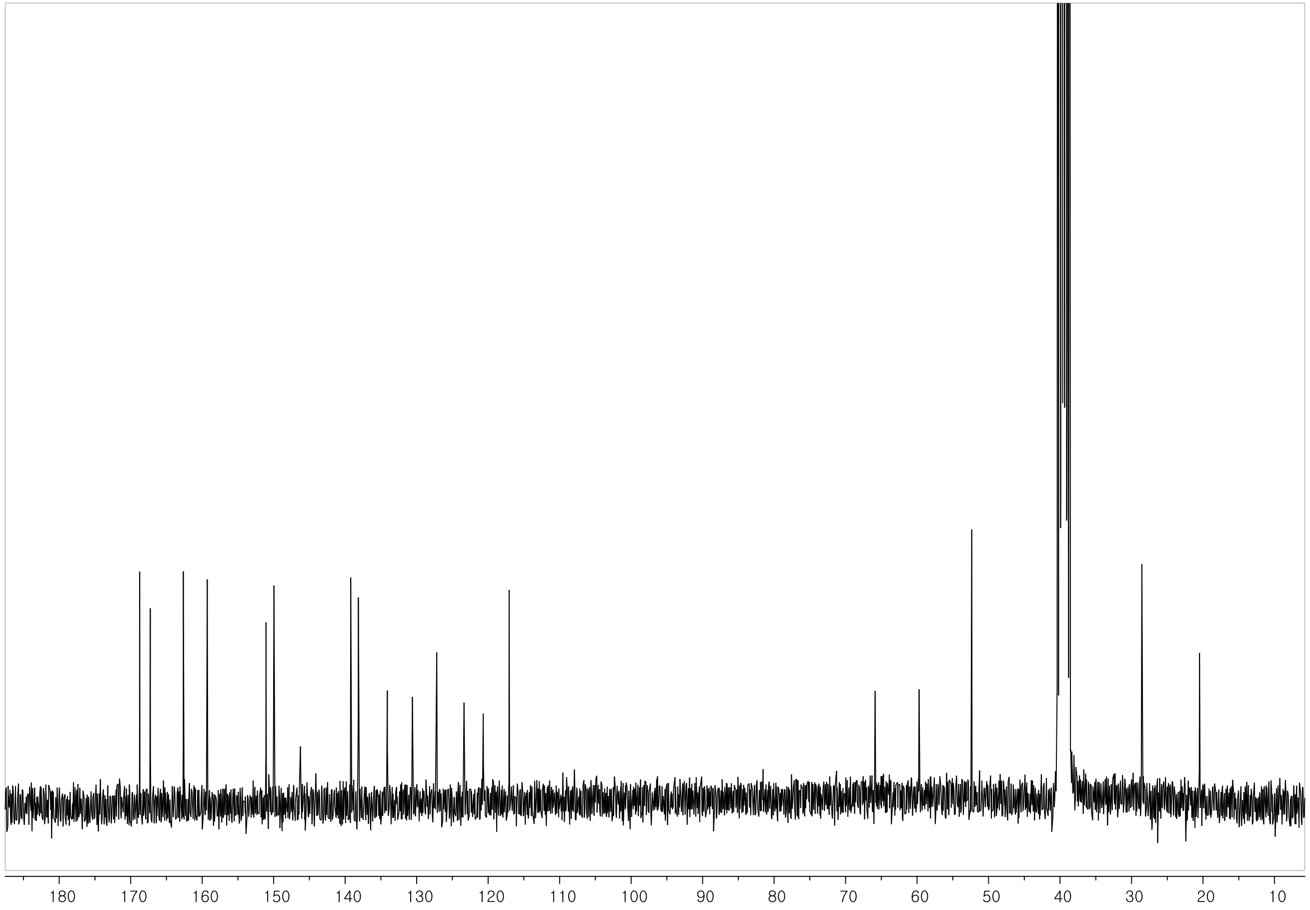

Figure S2. The ^{13}C NMR (150 MHz, DMSO-d_6) spectrum of terrelumamide A (**1**).

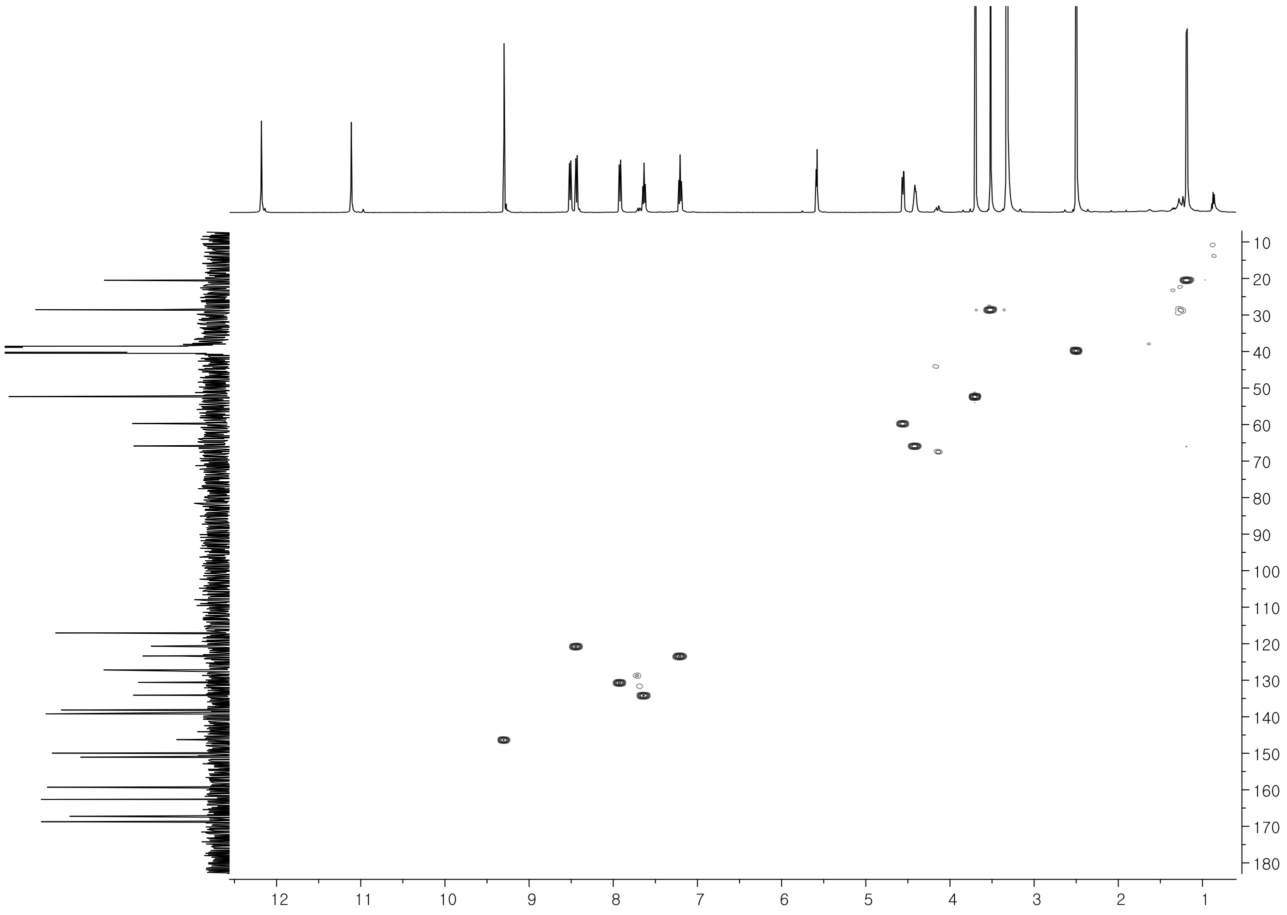

Figure S3. The HSQC (500 MHz, DMSO-d_6) spectrum of terrelumamide A (**1**).

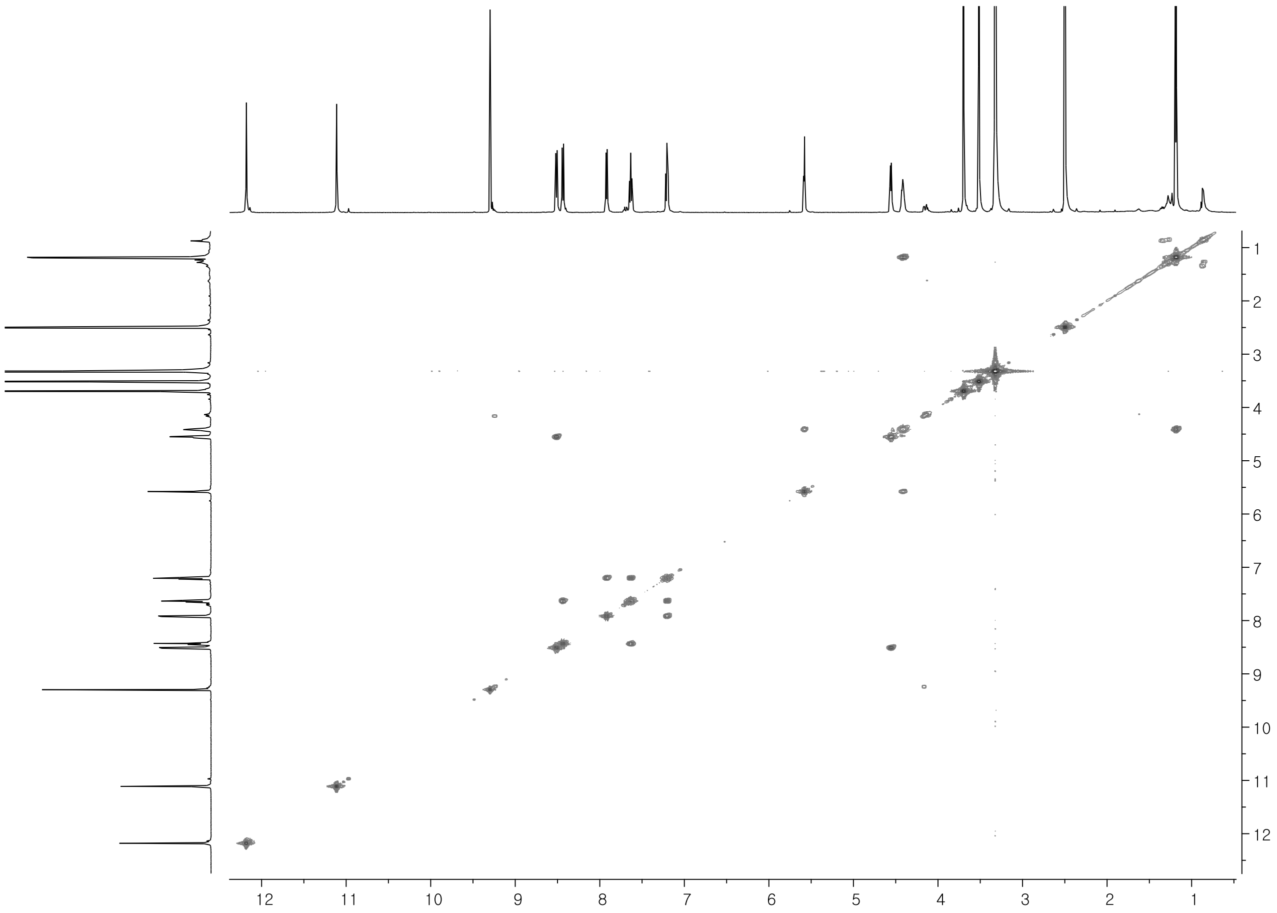

Figure S4. The COSY (500 MHz, DMSO-d_6) spectrum of terrelumamide A (**1**).

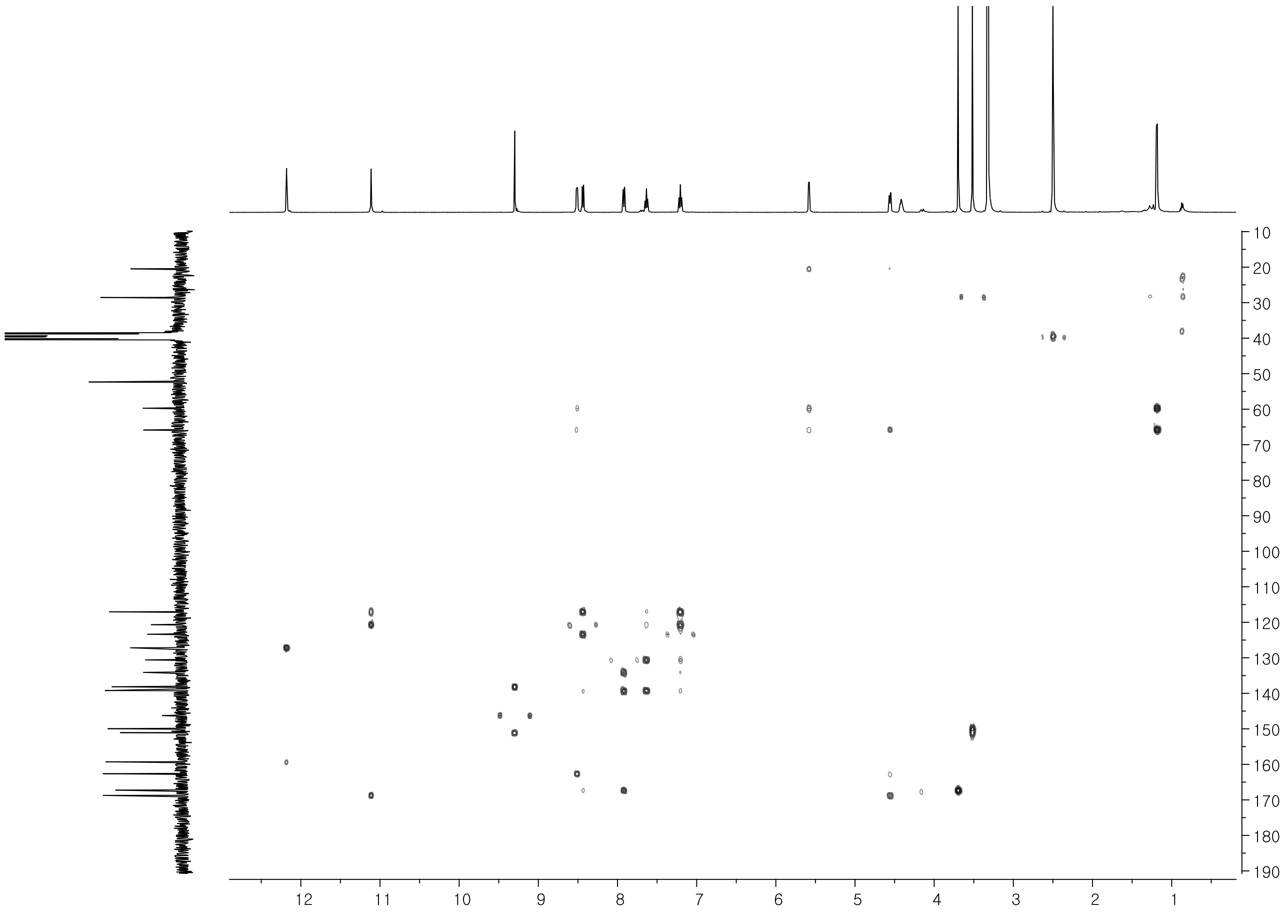

Figure S5. The HMBC (500 MHz, DMSO-d_6) spectrum of terrelumamide A (**1**).

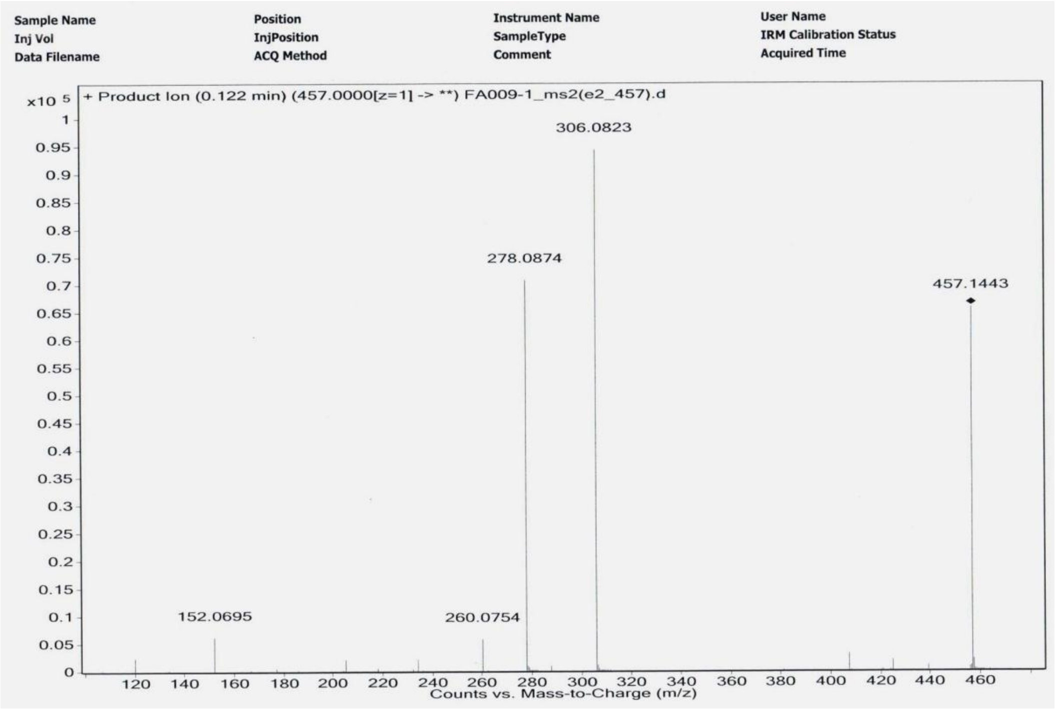

Figure S6. The ESI-Q-TOF-MS/MS spectrum of terrelumamide A (**1**).

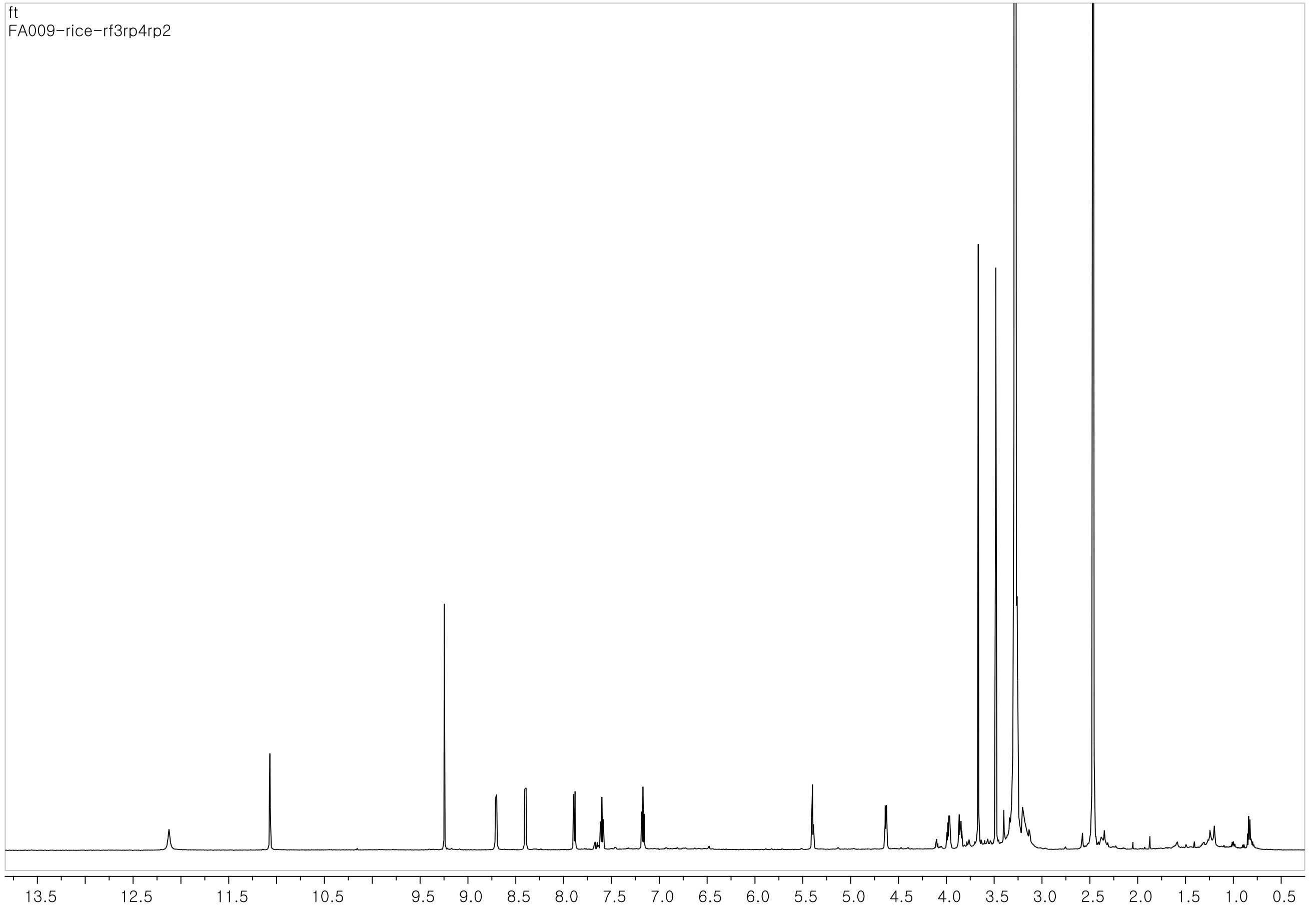

Figure S7. The ^{1}H NMR (500 MHz, DMSO-d_6) spectrum of terrelumamide B (**2**).

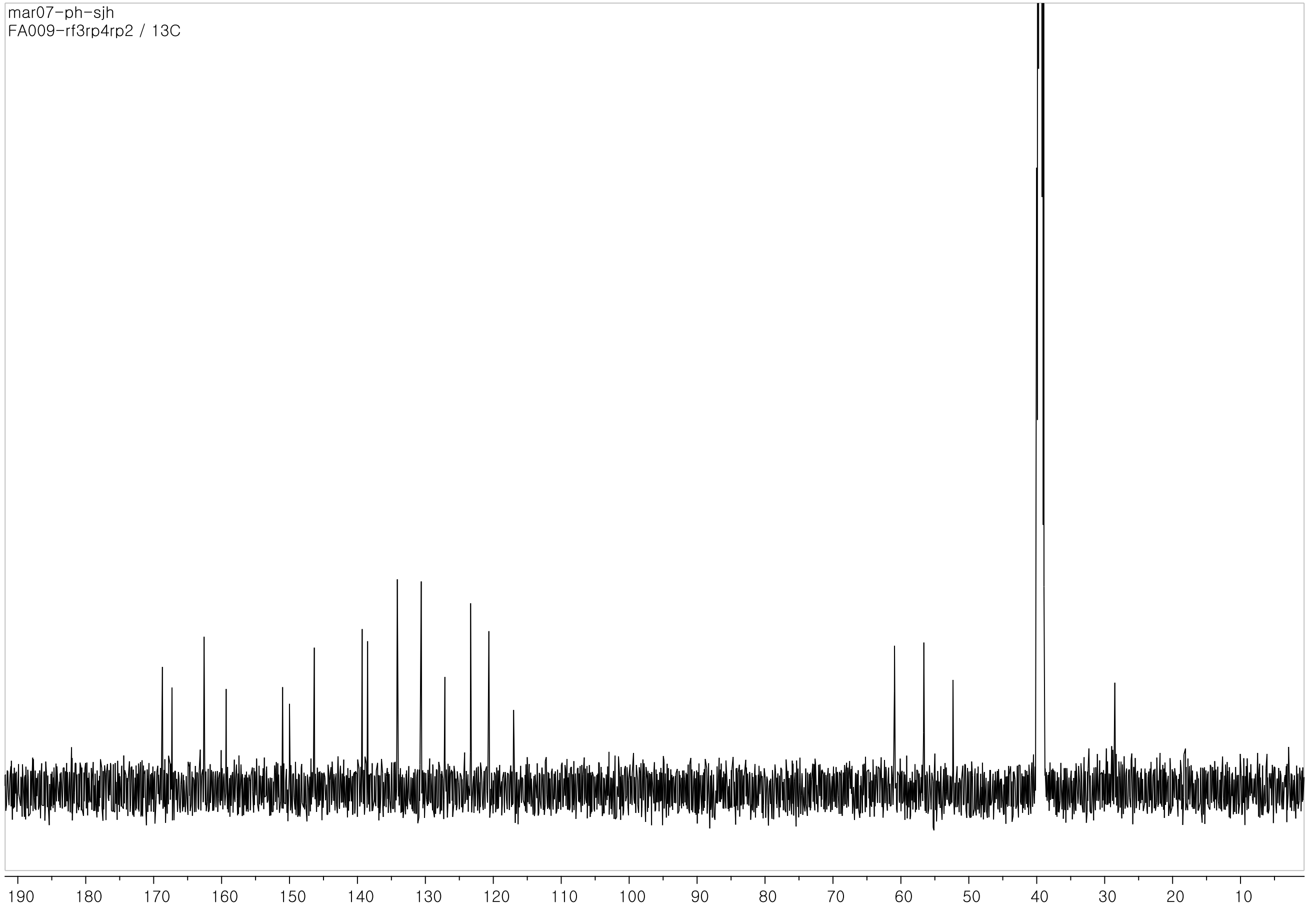

Figure S8. The ^{13}C NMR (125 MHz, DMSO-d_6) spectrum of terrelumamide B (**2**).

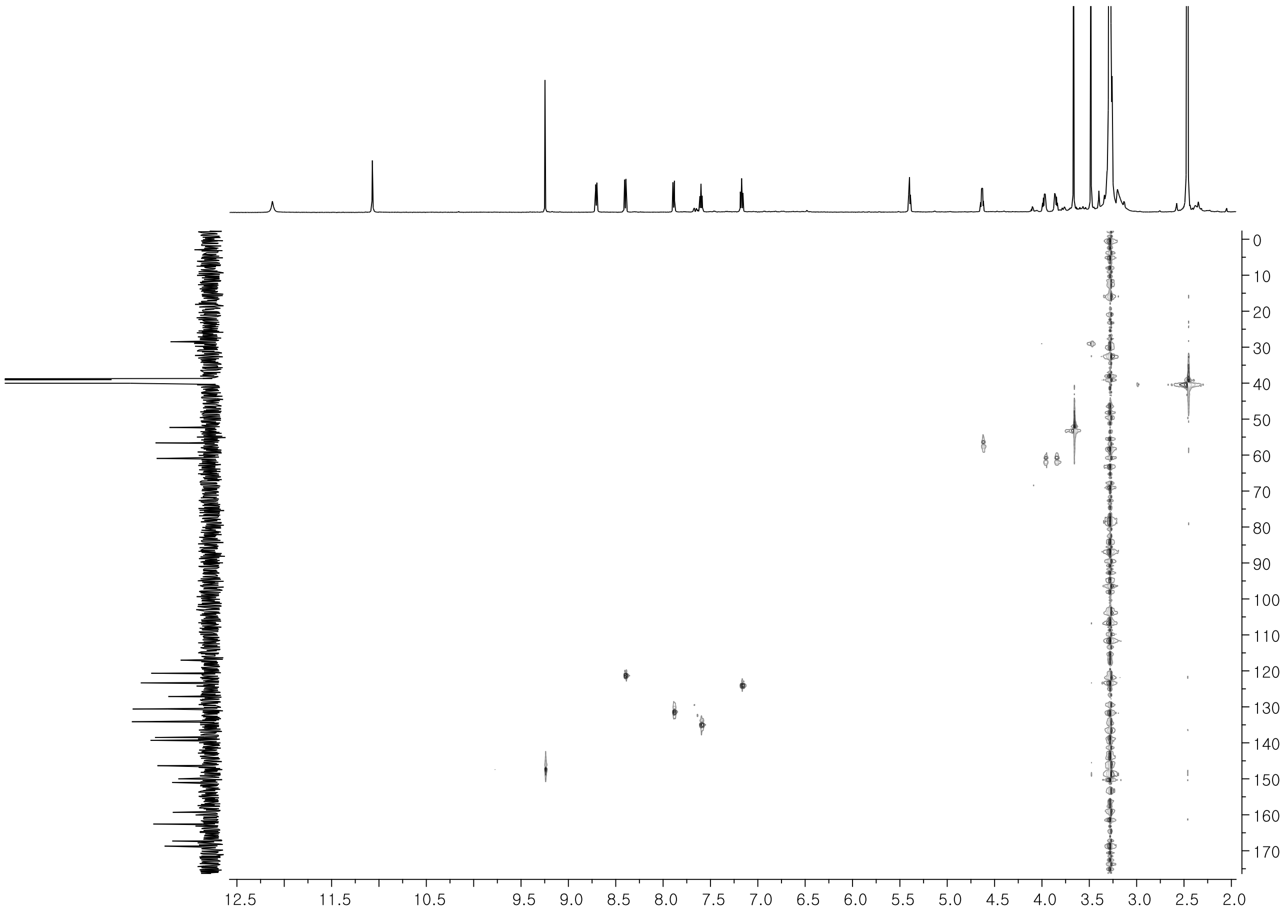

Figure S9. The HSQC (600 MHz, DMSO-d_6) spectrum of terrelumamide B (**2**).

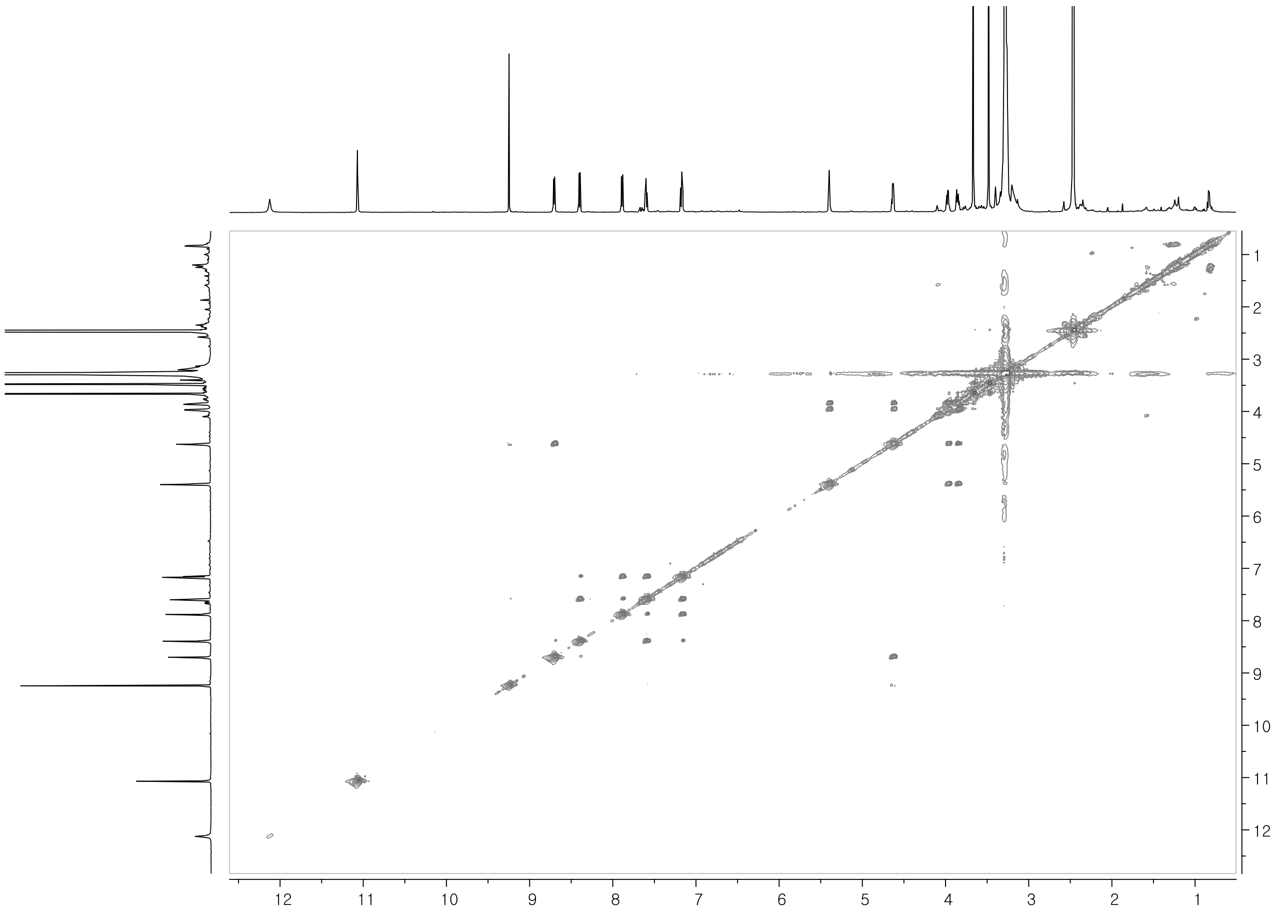

Figure S10. The COSY (600 MHz, DMSO-d_6) spectrum of terrelumamide B (**2**).

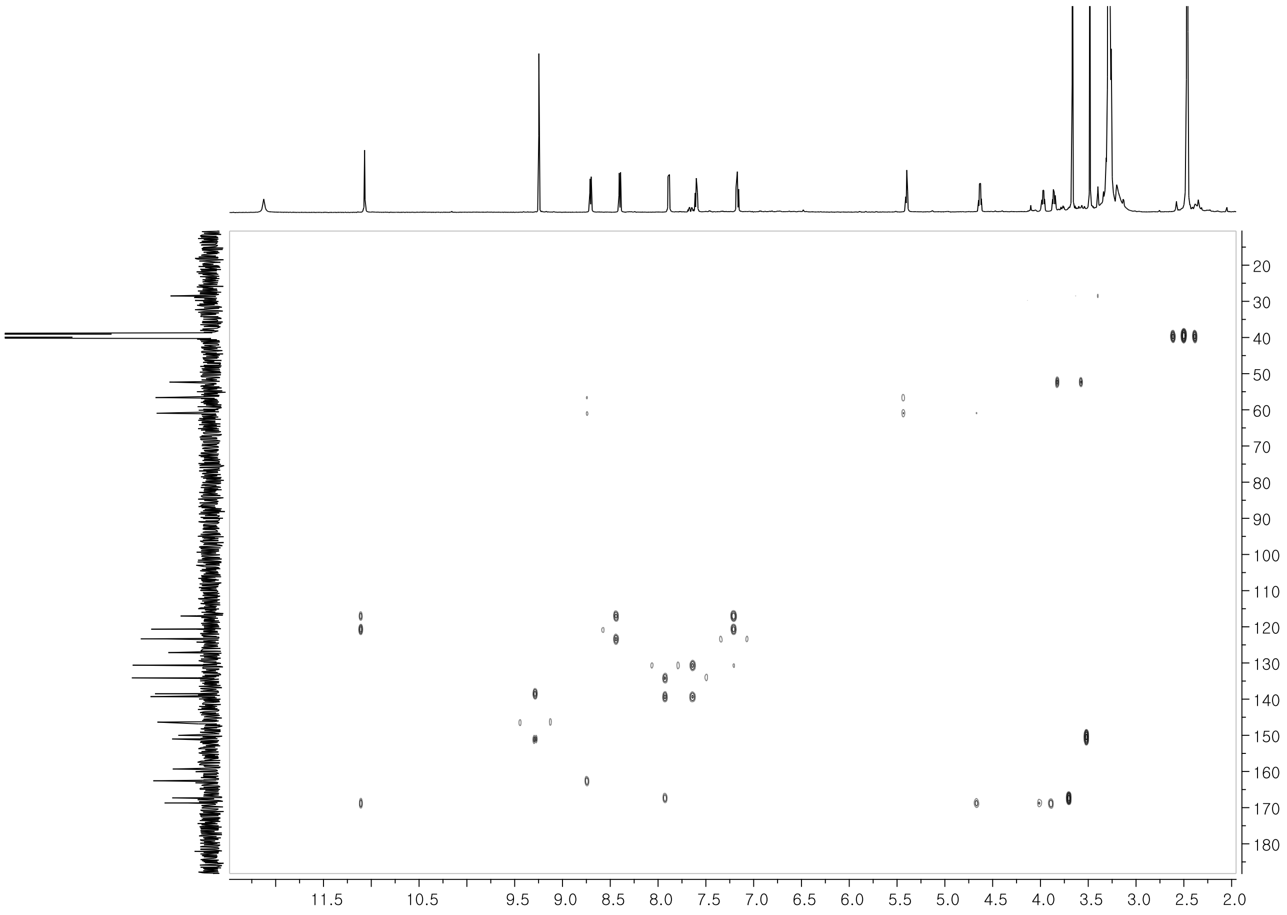

Figure S11. The HMBC (600 MHz, DMSO-d_6) spectrum of terrelumamide B (**2**).

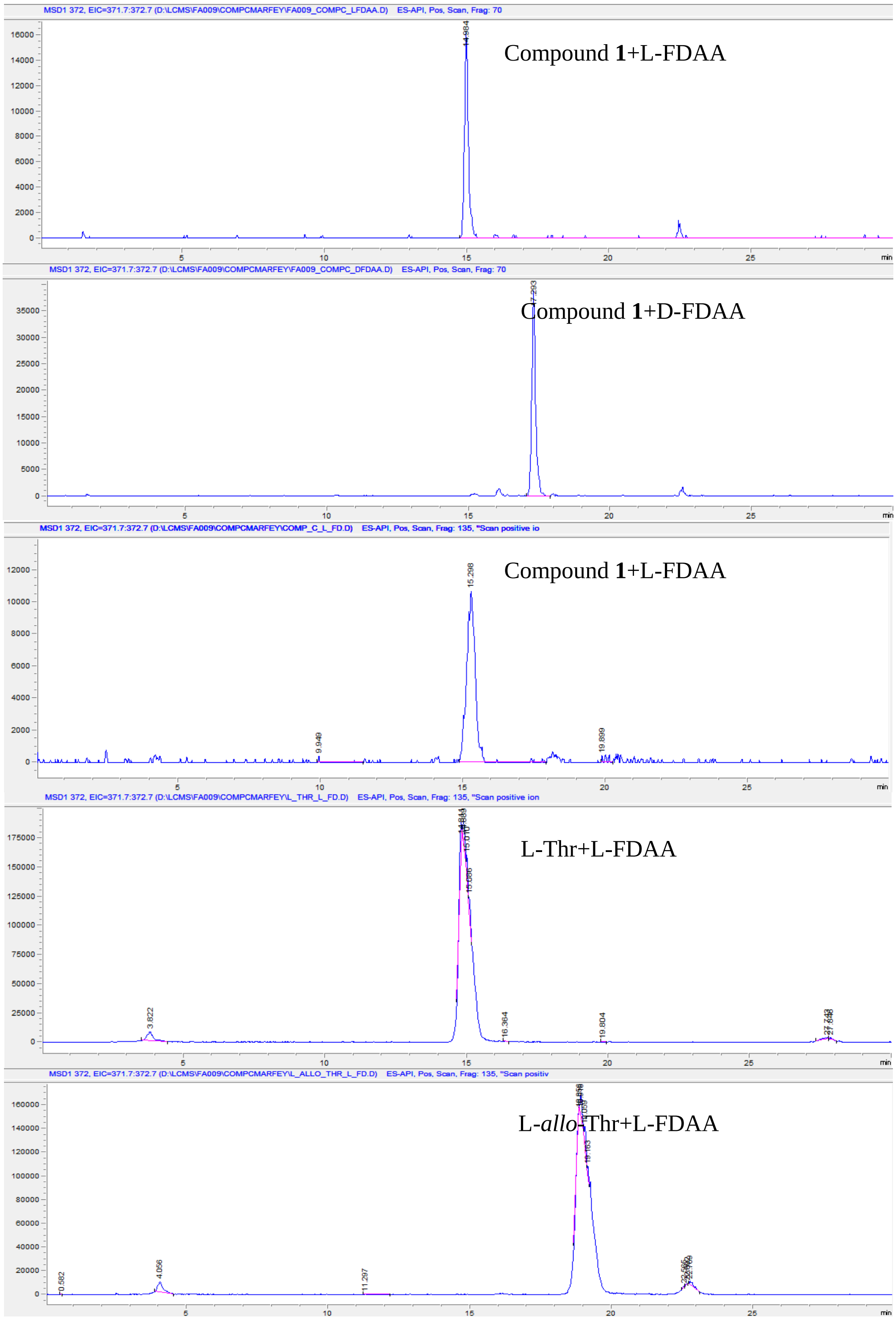

Figure S12. The results of advanced Marfey's method of terrelumamide A (**1**).

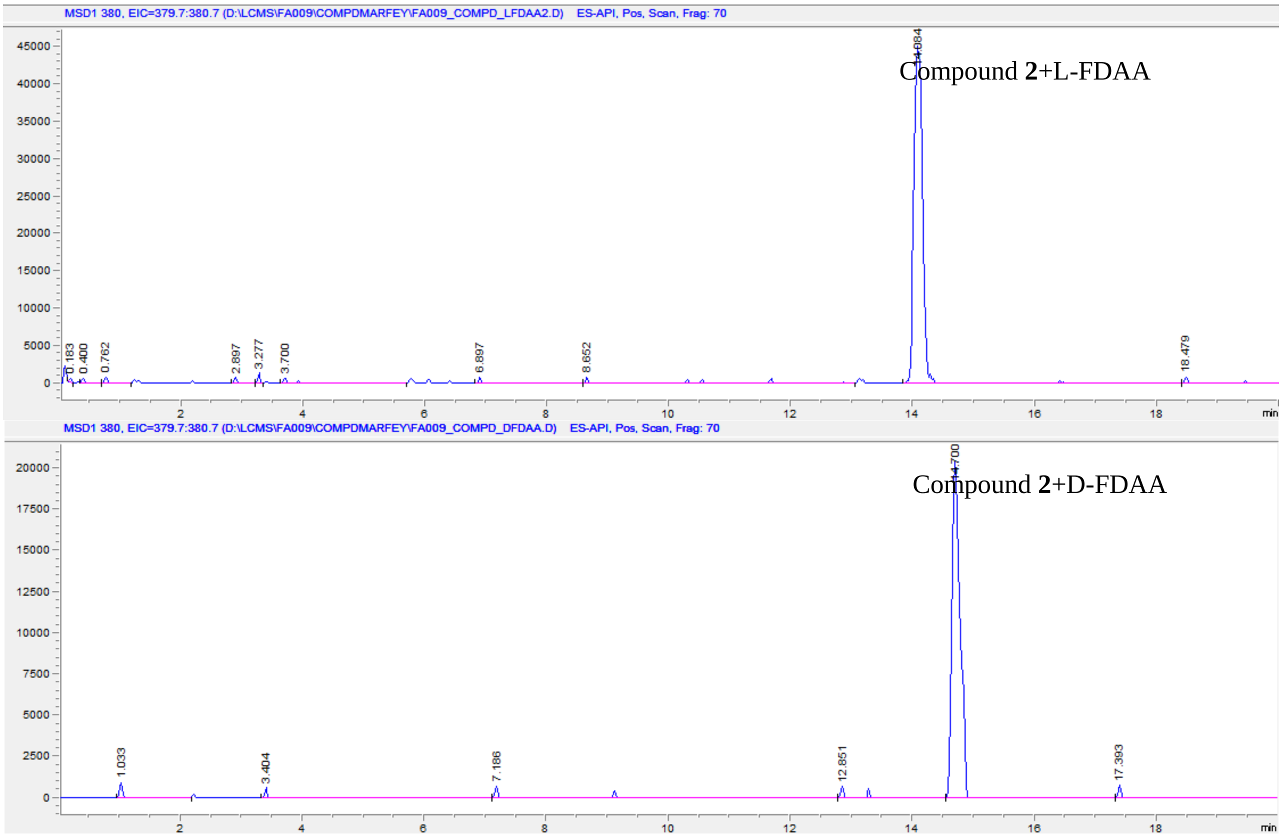

Figure S13. The results of advanced Marfey's method of terrelumamide B (**2**).

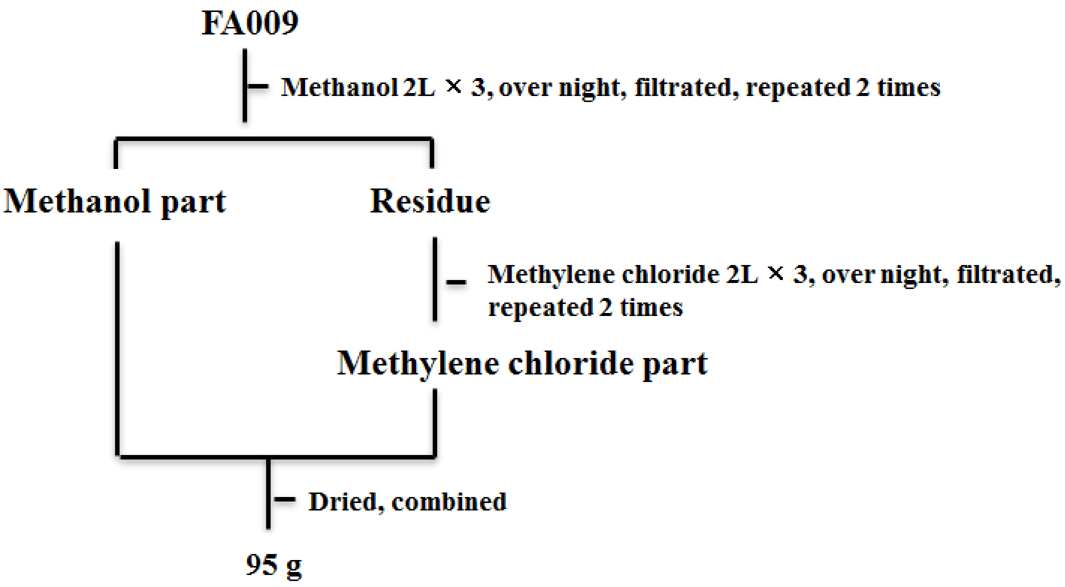

Figure S14. The work flow sheet of extraction procedure.

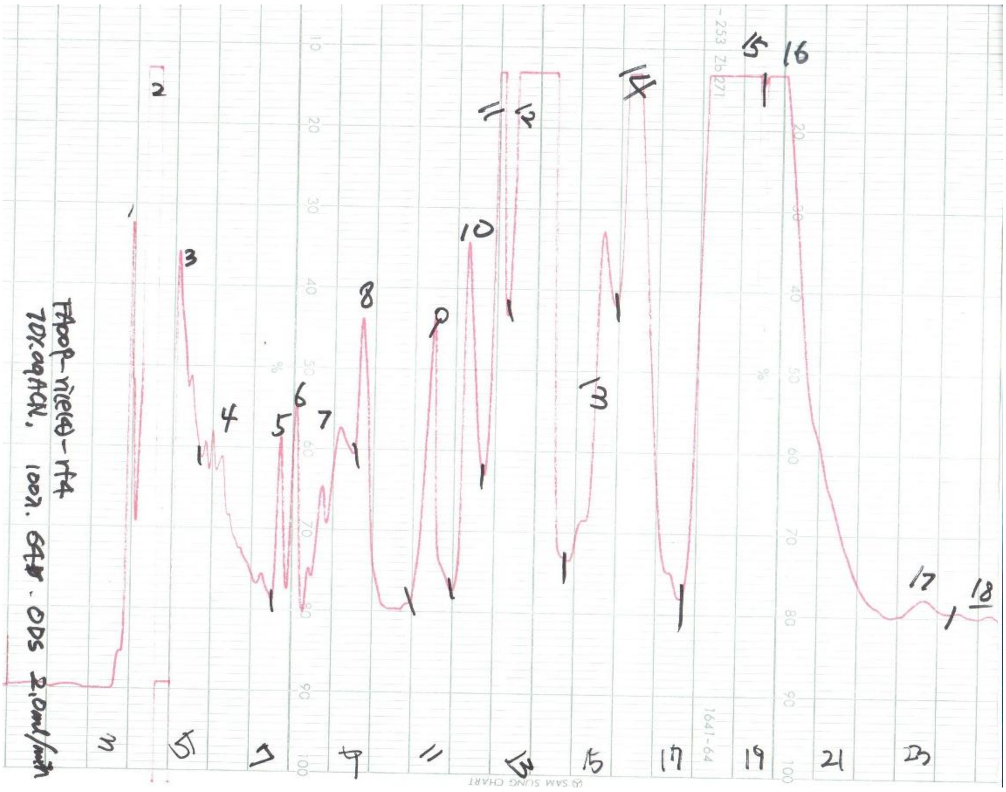

Figure S15. The LC chart of isolation.

References

1. Shin, J.H.; Shin, D.W.; Noh, M. Interleukin-17A inhibits adipocyte differentiation in human mesenchymal stem cells and regulates pro-inflammatory responses in adipocytes. *Biochem. Pharmacol.* **2009**, *77*, 1835–1844.

© 2015 by the authors; licensee MDPI, Basel, Switzerland. This article is an open access article distributed under the terms and conditions of the Creative Commons Attribution license (http://creativecommons.org/licenses/by/4.0/).
